# Real-world use of ustekinumab therapeutic drug monitoring in moderate to severe psoriasis

**DOI:** 10.3389/fmed.2022.1017323

**Published:** 2022-12-08

**Authors:** Laida Elberdín, Rosa M. Fernández-Torres, María Mateos, María Outeda, Eva Blanco, María I. Gómez-Besteiro, Isabel Martín-Herranz, Eduardo Fonseca

**Affiliations:** ^1^Department of Pharmacy, Complexo Hospitalario Universitario de A Coruña (CHUAC), Sergas, Instituto de Investigación Biomédica de A Coruña (INIBIC), Universidade da Coruña (UDC), A Coruña, Spain; ^2^Department of Dermatology, Complexo Hospitalario Universitario de A Coruña (CHUAC), Sergas, Instituto de Investigación Biomédica de A Coruña (INIBIC), Universidade da Coruña (UDC), A Coruña, Spain; ^3^Clinical Epidemiology and Biostatistics Unit, Complexo Hospitalario Universitario de A Coruña (CHUAC), Sergas, Instituto de Investigación Biomédica de A Coruña (INIBIC), Universidade da Coruña (UDC), A Coruña, Spain

**Keywords:** ustekinumab, psoriasis, therapeutic drug monitoring, biologic therapy, real-world

## Abstract

**Introduction:**

There is growing evidence that therapeutic drug monitoring of biologic therapy is beneficial in psoriatic patients. With respect to ustekinumab, the available evidence has not shown any relationship yet. The objective of this study is to identify correlations among ustekinumab trough concentrations, anti-ustekinumab antibodies and clinical response in moderate-to-severe plaque psoriasis patients, in a real-world setting.

**Methods:**

Observational prospective follow-up study in psoriatic patients treated with ustekinumab. Patients were classified in optimal (PASI ≤ 3) and suboptimal responders (PASI > 3). Mann–Whitney *U* test and Spearman’s rank correlation coefficient were used. Receiver-operator characteristic curve analysis was performed to identify ustekinumab concentration cut-off to achieve optimal response. A *p*-value < 0.05 was considered statistically significant.

**Results:**

A total of 59 patients were included. Forty-eight patients (81.4%) corresponded to optimal responders and 11 (18.6%) to suboptimal responders. There was significant difference to ustekinumab concentrations: 0.7 μg/mL (range <0.1–1.8) vs. 0.4 μg/mL (range <0.1–0.8) respectively (*p* = 0.007). Positive correlation between ustekinumab concentration and psoriasis area and severity index (PASI) value was detected (*p* = 0.009). A cut-off value of 0.6 μg/mL ustekinumab concentration was found to achieve clinical response. Anti-ustekinumab antibodies were detected in 2 (3.4%) samples, both suboptimal responders.

**Conclusion:**

A positive correlation exits between ustekinumab concentration and clinical response (optimal response PASI values ≤ 3) in blood draws performed before drug administration. The measurement of anti-ustekinumab antibodies could be considered in treatment failure.

## Introduction

Psoriasis is a chronic, inflammatory skin disease recognized by the World Health Organization as a major global health problem. This disease affects 2–4% of the population (1). Psoriasis treatment has developed with the appearance of biological agents. These biologics target key mediators such as tumor necrosis factor-a, interleukin (IL)-12/23, or IL-17A (2). Biologics have been shown to be highly effective for naïve patients. Nevertheless, the response may decrease over time and lead to treatment discontinuation/switching. Lack of effectiveness is the most common reason for stopping biologics (3).

Ustekinumab is a human monoclonal antibody targeted on the p40 subunit that is shared by interleukins 12 and 23. Its efficacy has been studied in the pivotal PHOENIX trials (2, 4). The few real-world studies have demonstrated the maintenance of long-term efficacy of ustekinumab treatment in moderate-severe plaque psoriasis in daily practice, even for 8 years (5). Nevertheless, many patients do not respond or lose response during ustekinumab treatment.

Therapeutic drug monitoring (TDM) aids in clinical decision making. It consists of measuring the serum concentrations of drugs and/or anti-drug antibodies in clinical practice. (6). In inflammatory bowel disease or rheumatoid arthritis, it has been demonstrated that adequate biologicals serum concentrations are associated with a good clinical response. Mounting evidence shows that TDM of biopharmaceuticals in psoriasis is beneficial too (7). However, studies on the immunogenicity and the clinical relevance of TDM of ustekinumab in psoriatic patients are scarce. This practice is not yet fully implemented, as is the case for tumor necrosis factor-a (adalimumab, etanercept, or infliximab) (7).

This study evaluates the correlation between ustekinumab trough concentrations and anti-ustekinumab antibodies (AUAs) with clinical response in patients with moderate to severe plaque psoriasis, in a real-world setting.

## Materials and methods

### Study design and patients

Observational prospective follow-up study. Patients with moderate to severe plaque type psoriasis treated with ustekinumab by Department of Dermatology of University Hospital of La Coruña (Spain) from September 2017 to March 2022 were included. Ustekinumab dosage was 45 mg every 12 weeks if patient weight was <100 kg, and 90 mg every 12 weeks if weight was ≥100 kg, after administration drug in 0 and 4 weeks (dosage regimen induction). Ustekinumab injections were administered during follow-up visits ensuring adherence to treatment. Patients weighing <100 kg could modify the dose to 90 mg every 12 weeks if they did not achieve a good response. Patients were candidates for inclusion in the study if they were at least 18 years old and treated with ustekinumab for ≥6 months.

### Measurement of serum ustekinumab and anti-ustekinumab antibodies levels

The serum samples for measurement ustekinumab and AUAs concentration were drawn immediately prior to ustekinumab administration during routine clinics visits.

The collection, preparation of serum samples and quantification of drug and anti-drug antibodies were performed as in our etanercept and anti-etanercept antibody quantification study (8). Free ustekinumab and AUAs concentration were determined using enzyme linked immunosorbent assay (ELISA; PROMONITOR^®^-UTK and PROMONITOR^®^-anti-UTK method, Grifols^®^, Derio, Spain). PROMONITOR^®^-UTK and PROMONITOR^®^-anti-UTK test validated by Shankar white paper and FDA guidelines (8). The ustekinumab assay has a limit of quantification of 0.132 μg/mL and a lower limit of detection of ≤0.111 μg/mL. With regard to AUAs, the assay has a quantification limit of 3 AU/mL and a minimum detection limit of 1.9 AU/mL. AUAs were considered positive when the concentration exceeded 3 AU/mL.

### Clinical response

Psoriasis area and severity index (PASI) was evaluated at baseline and at extractions time. Clinical response to ustekinumab treatment was assessed using absolute PASI at the extraction time of serum sampling. Optimal response was defined to PASI values ≤ 3, according to our previous studies (5, 9), and treatment goal (Physician’s Global Assessment of clear or nearly clear and/or PASI < 2) in the British Association of Dermatologists’ guidelines for biologic therapy for psoriasis and practical update of the recommendations published by the psoriasis group of the Spanish Academy of Dermatology and Venereology on the treatment of psoriasis with biologic therapy (1, 10).

The failure of biological therapies was classified into primary failure, secondary failure, and side effects, according to our previous studies (5, 8, 11, 12).

Based on the efficacy of ustekinumab treatment, patients were classified into two groups: optimal responders (PASI ≤ 3) and suboptimal responders (PASI > 3).

### Ethics and authorizations

The Ethic Committee for Clinical Investigation of Galicia (Spain) approved this study (Protocol Code 2017/378). It was classified as postauthorization prospective study by the Agencia Española del Medicamento y Productos Sanitarios (Protocol code EFP-FAR-2017-01). All patients provided written informed consent before inclusion in the study. This study was conducted in accordance with the Helsinki Declaration of 1964 and its later amendments.

### Statistics

Statistical analyses were conducted with SPSS statistics version 24.0. Differences between groups were analyzed by Mann–Whitney *U* test. Correlations between ustekinumab and AUAs concentration and PASI value were assessed using Spearman’s rank correlation coefficient. Receiver-operator characteristic (ROC) curve analysis was performed to identify ustekinumab concentration cut-off to achieve optimal response. A *p*-value < 0.05 was considered statistically significant.

## Results

### Patient characteristics

All patients with moderate to severe psoriasis treated at our hospital who met the inclusion criteria were included, a total of 59 patients were enrolled. The psoriatic arthritis comorbidity was diagnosed in 13.6%, patients displaying mainly dermatological symptoms. Most patients received a 45 mg ustekinumab dose. Four patients (6.8%) received a 90 mg ustekinumab dose despite weighing <100 kg due to insufficient response. Only one patient was co-treated with methotrexate. Forty-seven patients (79.7%) had previously been treated with at least one biological (Table 1).

**TABLE 1:** Demographic and clinical characteristics of the study population.

Demographic characteristics	
Male [*n* (%)]	39 (66.1)
Weight in kg [median (range)]	80.0 (50.0–128.0)
Body mass index in Kg/m^2^ [median (range)]	27.7 (19.0–47.0)
**Clinical characteristics**	
**Diagnosis [*n* (%)]**	
Psoriasis	51 (86.4)
Psoriatic arthritis	8 (13.6)
Age at diagnosis in years [median (range)]	30.0 (3.0–73.0)
PASI at baseline [median (range)]	11.8 (2.7–35.6)
BSA at baseline [median (range)]	15.0 (3.0–67.0)
Initial PASI ustekinumab treatment [median (range)]	10.8 (0.0–35.6)
Initial BSA ustekinumab treatment [median (range)]	12.5 (0.0–60.0)
**Ustekinumab treatment**	
Duration in months [median (range) Dosage]	33.3 (6.0–142.7)
45 mg [*n* (%)]	44 (74.6)
90 mg [*n* (%)]	15 (25.4)
Patients who had previously received other biological drugs and reason of discontinuation [*n* (%)]	47 (79.7)
Etanercept	42 (89.4)
Primary failure	19 (45.2)
Secondary failure	15 (35.7)
Adverse events	5 (11.9)
Others	3 (7.2)
Adalimumab	17 (36.2)
Primary failure	3 (17.6)
Secondary failure	10 (58.8)
Anti-adalimumab antibodies	4 (23.6)
Infliximab	5 (10.6)
Primary failure	1 (20.0)
Secondary failure	1 (20.0)
Anti-infliximab antibodies	2 (40.0)
Adverse events	1 (20.0)
Secukinumab	3 (6.4)
Primary failure	0 (0.0)
Secondary failure	3 (100.0)
Efalizumab	2 (4.3)
Primary failure	1 (50.0)
Secondary failure	0 (0.0)

### Relationship between ustekinumab and anti-ustekinumab antibodies concentrations and clinical response

Patients were divided into two efficacy groups at extraction sample moment: 48 (81.4%) were optimal responders (PASI ≤ 3) and 11 (18.6%) were suboptimal responders (PASI > 3). In the group of optimal responders, 83.3% of patients had PASI 0, 85.4% PASI ≤ 1, and 93.8% PASI ≤ 2. Table 2 summarizes the clinical characteristics of efficacy groups.

**TABLE 2:** Clinical characteristics of the two efficacy groups: optimal (PASI ≤ 3) and suboptimal (PASI > 3) responders.

	Optimal responders	Suboptimal responders	*p*
Total patients [*n* (%)]	48 (81.4)	11 (18.6)	–
PASI at baseline [median (range)]	11.3 (2.7–35.6)	23.0 (9.0–28.5)	0.112
PASI at initial ustekinumab treatment [median (range)]	10.3 (0.0–35.6)	14.4 (7.6–34.2)	0.036
Treatment time with ustekinumab, months [median (range)]	47.2 (6.0–142.7)	15.2 (6.5–58.3)	0.009
**Ustekinumab dosage**			
45 mg [*n* (%)]	38 (79.2)	6 (50.5)	0.094
90 mg [*n* (%)]	10 (20.8)	5 (45.5)	
Weight in kg [median (range)]	78.0 (50.0–117.0)	88.0 (71.0–128.0)	0.044
Body mass index (BMI) (Kg/m^2^) [median (range)]	27.5 (19.0–38.5)	27.4 (19.0–47.0)	0.086
Ustekinumab dose/body-weight (mg/kg) [median (range)]	0.7 (0.5–1.2)	0.6 (0.5–1.0)	0.992
PASI at the extraction sample time [median (range)]	0.0 (0.0–3.0)	6.0 (4.0–21.9)	0.000
Ustekinumab concentration (μg/mL) [median (range)]	0.7 (<0.1–1.8)	0.4 (<0.1–0.8)	0.007
Samples positive anti-ustekinumab antibodies [*n* (%)]	0 (0.0)	2 (18.2)	0.003

**FIGURE 1:**
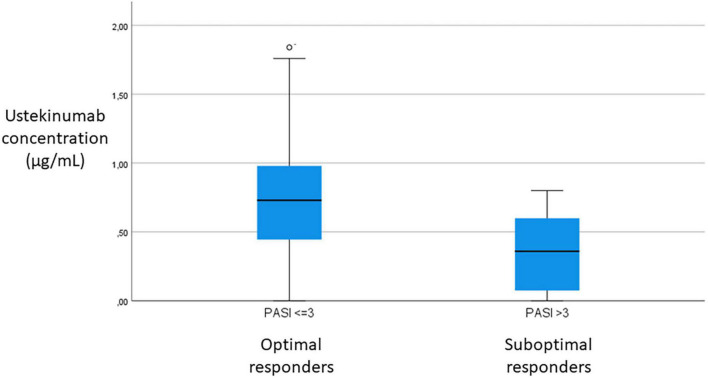
Comparison of ustekinumab concentration between optimal (PASI ≤ 3) and suboptimal (PASI > 3) responders.

**FIGURE 2:**
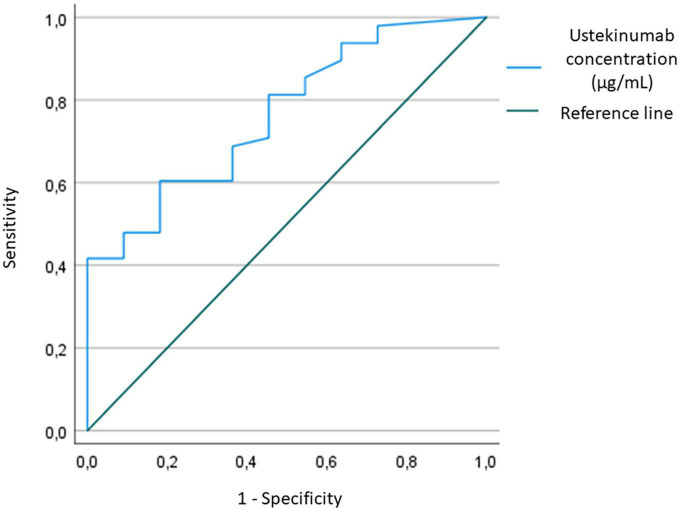
Receiver-operator characteristic (ROC) curve for sensitivity and specificity to determine ustekinumab concentration cut-off to achieve optimal response.

There was no significant difference between efficacy groups in demographic and clinical characteristics, except for PASI at initial ustekinumab treatment values (*p* = 0.036), treatment time with ustekinumab treatment (*p* = 0.009) and body-weight (*p* = 0.044). Suboptimal responders group had higher PASI at initial ustekinumab treatment and body-weight than optimal responders. Median ustekinumab concentrations were higher for optimal responders than for suboptimal: 0.7 μg/mL (range <0.1–1.8) vs. 0.4 μg/mL (range <0.1–0.8) (*p* = 0.007). Positive correlation between ustekinumab concentration and PASI value (*p* = 0.009) was detected. To determine the lower ustekinumab concentration to achieve clinical response (PASI ≤ 3), we designed a ROC curve. We have found a cut-off value of 0.6 μg/mL ustekinumab concentration (sensibility 60%, specificity 81%) (Figures 1, 2).

Anti-ustekinumab antibodies were detected in only two samples (3.4%), both patients in suboptimal responders’ group. In the first patient, 8.6 UA/ml and no ustekinumab concentration were detected at 9.3 months of treatment, with a PASI value at extraction time of 8.0. This patient had been previously treated with etanercept and adalimumab developing secondary failure. In the second patient, at 6.7 months of treatment and with a PASI value 21.9, 14.0 UA/ml and no ustekinumab concentration were detected. This patient had been previously treated with secukinumab and etanercept developing secondary and primary failure respectively.

## Discussion

Ustekinumab is an effective treatment to psoriasis. Most of patients included in our study showed an optimal response, as in literature (2–5, 11, 13, 14). In our study, a positive association was found between serum concentrations of ustekinumab and optimal response in psoriatic patients. Patients with AUAs titles and without ustekinumab concentrations were no-responders to treatment.

The findings of this study are in line with previous studies (15–17). In the study of Toro-Montecinos et al. (15), 54 serum samples from 27 psoriatic patients that were receiving ustekinumab for at least 24 weeks were analyzed. They measured serum ustekinumab levels at weeks 6 and 12. PASI score was determined at week 12 just before the next ustekinumab dose. As in our study, they used absolute PASI score to assess clinical response. No correlation was found between serum drug levels and absolute PASI at week 12. However, an inverse linear correlation was found between absolute PASI and drug levels measured at week 6 (*p* = 0.0001). Ustekinumab levels at week 6 in patients with excellent response (PASI ≤ 3) was higher then patients with PASI > 3 (1.144 vs. 0.54 μg/mL, *p* = 0.0067). However, the authors state that this conclusion must be confirmed prospectively in clinical practice with a larger number of patients. In Tsakok et al. (16) study, most of samples were collected without reference to treatment administration. The median time from last dose was 28 days [interquartile range (IQR), 16–57 days; range, 0–98 days; data available for 515 samples]. Martín-González et al. (17) proposed a therapeutic range of ustekinumab concentration for moderate and good responders. Nevertheless, their study only includes samples at 52 weeks of 37 patients, and the division of patients into response groups is not shown.

On the other hand, some studies did not find a correlation between ustekinumab concentrations and clinical response (18, 19). Menting et al. (18) included 41 psoriatic patients treated with ustekinumab. Patients were divided into three groups: no-responders (< PASI50), moderate responders (PASI50-PASI75) and good responders (≥ PASI75). No correlation was found at weeks 16 and 28 between clinical response and ustekinumab levels. In the multicenter study of De Keyser et al. (19) 229 samples of 137 psoriatic patients were analyzed. Serum samples and PASI values were obtained at baseline and at weeks 16, 28, 40, 52, or ≥64 of ustekinumab treatment. Patients were classified according clinical response as in Menting et al. (18) study. Once again authors did not find differences in serum concentrations between responders’ groups and no significant correlation was found between ustekinumab trough levels and mean change in PASI. But it should be noted that both studies used PASI variance rather than absolute PASI.

A strength of this study is that we classified patients into two groups based on absolute PASI value: optimal responders (PASI ≤ 3) and suboptimal responders (PASI > 3). We choose this cut-off point since, just as we proposed in 2012 (9), in a real-world setting, the maintenance of low PASI index may be a most useful approach for patients and physicians than the reduction of the initial values, when the remaining lesions continue to have clinical significance. This is especially useful when several treatments are chained in succession, without washing periods, something common in clinical practice. Recently, Mahil et al. (1) analyzed data from 13,422 patients and they concluded that an absolute PASI ≤ 2 corresponds with PASI 90 response and is a relevant disease end point for treat-to-target approaches in psoriasis.

The prevalence of AUAs in our study was 3.4%, which is comparable to the prevalence rates reported in literature (1–11%) (18–20). The two patients with presence of AUAs and without ustekinumab concentrations were no-responders to treatment. These data are consistent with other published studies, which suggest a trend toward decreased response to treatment with the formation of AUAs. However, we cannot conclude a correlation between AUAs and non-response to treatment given the low number of patients in our study with AUAs titers.

Hermans et al. (7) reviewed the literature on TDM of biopharmaceuticals in the treatment of psoriasis. They found that ustekinumab studies were particularly scarce, because monitoring serum concentrations of ustekinumab and AUAs had not yet been fully implemented, as in the case for adalimumab or infliximab. They concluded that further research was needed. Our findings contribute to broaden the knowledge in this field.

Our study has some limitations. First, is a single-center observational prospective follow-up study. Although multi-center studies required to support changes in clinical practice, we considered that this is a study conducted well-designed. Data from this study could be used to design larger confirmatory studies.

Second, the small number of patients included. Anyway, the sample used in the study is representative.

Third, the data were collected at different time points, because it analyses a real-world environment.

Another limitation is the association of serum ustekinumab levels are used to assess the clinical response without taking into account the expression of any marker. For example, the presence of the Human Leukocyte Antigen (HLA)-C*06 allele has been associated as a potential predictor of ustekinumab response in psoriasis (19, 21–23). However, De Keyser et al. (19) study and Van Vugt et al. (24) meta-analysis conclude that there is no justification for excluding patients with negative HLA-C*06 for ustekinumab treatment. Analyzing the expression patterns of the TGF-β gene and transcription activity profiles of TNF-α, TNFR1, and TNFR2 may also be useful for monitoring ustekinumab therapy (25, 26).

On the other hand, there are several scales to assess effectiveness: PASI, Dermatology Life Quality Index (DLQI) and Body Surface Area (BSA) (26, 27). In our study, only absolute PASI value was used, because it is the most useful criteria to assess whether the patient is within the therapeutic response parameters at any time (1, 10). The DLQI is the most widely used index to assess health-related quality of life in dermatology and psoriasis. Although easy to use and sensitive to change, this index has the limitation of one-dimensional structure and variable cross-cultural equivalence (10), so we have not used it.

All this limitations of our study could be taken to overcome the limitations in future studies.

This study adds a treatment strategy based in ustekinumab and AUAs levels in psoriatic patients. A lack of information on TDM for ustekinumab in psoriasis has been identified during the manuscript development. This study provides more evidence to the few available. Future studies should focus on understanding the TDM for ustekinumab in patients with psoriasis in real life.

## Conclusion

This real world-setting study found a correlation between ustekinumab concentration and clinical response in blood draws performed before drug administration. Patients with AUA titles presented treatment failure, so AUA measurement could be considered in patients who not respond to ustekinumab.

## Data availability statement

The original contributions presented in this study are included in the article/supplementary material, further inquiries can be directed to the corresponding author.

## Ethics statement

The studies involving human participants were reviewed and approved by the Ethic Committee for Clinical Investigation of Galicia (Spain). The patients/participants provided their written informed consent to participate in this study.

## Author contributions

LE performed the material preparation, data collection and analysis, and wrote the first draft of the manuscript. All authors contributed to the study conception and design, commented on previous versions of the manuscript, and read and approved the final manuscript.
